# A comprehensive understanding of the biocontrol potential of *Bacillus velezensis* LM2303 against *Fusarium* head blight

**DOI:** 10.1371/journal.pone.0198560

**Published:** 2018-06-01

**Authors:** Liang Chen, Junying Heng, Suya Qin, Ke Bian

**Affiliations:** 1 Provincial Key Laboratory for Transformation and Utilization of Cereal Resource, College of Bioengineering, Henan University of Technology, Zhengzhou, China; 2 Collaborative Innovation Center for Grain Storage Security in Henan Province, Zhengzhou, China; Dong-A University, REPUBLIC OF KOREA

## Abstract

*Fusarium* head blight (FHB) mainly caused by *F*. *graminearum*, always brings serious damage to wheat production worldwide. In this study, we found that strain LM2303 had strong antagonist activity against *F*. *graminearum* and significantly reduced disease severity of FHB with the control efficiency of 72.3% under field conditions. To gain a comprehensive understanding of the biocontrol potential of strain LM2303 against FHB, an integrated approach of genome mining and chemical analysis was employed. The whole genome of strain LM2303 was obtained and analyzed, showing the largest number of genes/gene clusters associated with biocontrol functions as compared with the known biocontrol strains (FZB42, M75, CAU B946). And strain LM2303 was accurately determined as a member of the *B*. *velezensis* clade using the phylogenomic analysis of single-copy core genes. Through genome mining, 13 biosynthetic gene clusters(BGCs) encoding secondary metabolites with biocontrol functions were identified, which were further confirmed through chemical analyses such as UHPLC-ESI-MS, including three antifungal metabolites (fengycin B, iturin A, and surfactin A), eight antibacterial metabolites (surfactin A, butirosin, plantazolicin and hydrolyzed plantazolicin, kijanimicin, bacilysin, difficidin, bacillaene A and bacillaene B, 7-o-malonyl macrolactin A and 7-o-succinyl macrolactin A), the siderophore bacillibactin, molybdenum cofactor and teichuronic acid. In addition, genes/gene clusters involved in plant colonization, plant growth promotion and induced systemic resistance were also found and analyzed, along with the corresponding metabolites. Finally, four different mechanisms of strain LM2303 involved in the biocontrol of FHB were putatively obtained. This work provides better insights into a mechanistic understanding of strain LM2303 in control of FHB, reinforcing the higher potential of this strain as a powerful biocontrol strain agent (BCA) for FHB control. The results also provide scientific reference and comparison for other biocontrol strains.

## Introduction

*Fusarium* head blight (FHB), mainly caused by *F*. *graminearum*, is one of the most devastating diseases of wheat (*Triticum aestivum* L.) and other small grain cereal crops worldwide, causing the reductions in yield and quality as well as the accumulation of mycotoxins in grains [1–3]. Notably, more than 7 million hectares which account for 25% of total areas in China were infected by FHB [4]. Various control strategies have been used to suppress FHB, including chemical fungicides treatment and resistant cultivars breeding, but so far none of them are enough to control this disease [5]. Due to long-term overuse of chemicals in agriculture, a chain of serious problems has arisen, including increased pathogen resistance, chemical residues, environmental pollution and health hazards [1]. Nowadays, owe to the rare pollution potential and health hazards, biocontrol strains have been considered as one of the most promising alternatives to the chemicals and have been commercially developed as biofertilizers and/or BCAs in many countries [6, 7], especially *Bacillus* strains, which were widely used as BCAs to control various plant diseases [6, 7].

Biocontrol activity of microbial strain can be mediated by its secondary metabolites, and the more metabolites that the strain produces, the stronger the biocontrol efficiency is [8, 9]. But, the traditional approach of identifying and characterizing the secondary metabolites was inefficient [10]. Excitingly, the fast development of DNA sequencing technologies has revolutionized almost every aspect of biology including the biocontrol strain, and complete genomes of some important biocontrol strains were obtained [6, 11]. Subsequently, the new effective approach known as genome mining, has provided an effective access to rapidly mining the genetic data and characterize the secondary metabolites potential of the strains.

Strain LM2303 was effective in inhibiting the conidia germination and mycelia growth of *F*. *graminearum* [12]. To better insight into the biocontrol potential and the mode of action against FHB, this study conducted to (1) evaluate the control efficacy of strain LM2303 in suppressing FHB under field conditions, (2) sequence, and analysis the LM2303 genome, and reclassified the taxonomy of strain LM2303, (3) mining of secondary metabolite BGCs and confirm these productions, (4) identify and analyze genes/gene clusters responsible for plant colonization, plant growth promotion and induced systemic resistance, along with the corresponding metabolites.

## Materials and methods

### Strains and culture conditions

*F*. *graminearum* used in this study was isolated from wheat spikes infected by FHB, and was maintained on potato dextrose agar (PDA) slant at 4°C. The fungus was cultured for 5d at 26°C, 120 r/min in a 250mL flask containing 100mL of CMC medium (CMC-Na 15g, NH_4_NO_3_·7H_2_O 0.5 g, yeast extract 1g, H_2_O 1L) to produce conidia. Then conidia suspension was filtered through sterile filter paper to remove mycelia, and adjusted to 2×10^5^ spores/mL using a Hemocytometer.

Strain LM2303 was maintained on nutrient agar (NA) slant at 4°C, then inoculated into a 500mL flask containing 175mL of Landy medium (L-glutamic acid 5g, glucose 20g, yeast extract 1g, phenylalanine 2mg, MgSO_4_·7H_2_O 0.5g, KCl 0.5g, MnSO_4_ 5mg, CuSO_4_·5H_2_O 0.16mg, FeSO_4_·7H_2_O 0.15mg, KH_2_PO_4_ 1g, H_2_O 1L, pH7.0) to culture for 48h at 33°C, 170 r/min to produce the metabolites. And LBGM broth (Tryptone 10g, yeast extract 5g, Nacl 5g, glycerol 1%(v/v), MnSO_4_ 0.1mM, H_2_O 1L) was used for biofilm formation.

### Field trial

To evaluate the biocontrol efficacy of strain LM2303 against FHB, the field trial was conducted at Beisazhen Village, Muchang Town, Liuan, Anhui province, China (E31°88’, N116°54’), in 2016. The wheat cultivar Yangmai 20 was used. The experimental plots (15 m^2^/plot, random block, 400 heads/plot) consist of 4 rows (2 m/row and 0.2 m between rows). At the initial stage of wheat anthesis, 800 mL of bacterial culture broth (2×10^8^ cfu/mL), 1000-fold dilution of 50% Carbendazim (WP), or sterile water were sprayed towards wheat spikes and roots, respectively. And 300 mL conidia suspension of *F*. *graminearum* (2×10^5^ spores/mL) were sprayed two days later. The treatments (four replicates per treatment) were both applied in the late afternoon approximately 2h before sunset, by spraying through a commercial sprayer consisting of 5 linear sprinklers and a CO_2_ pressure source. The sprayer was adjusted to 40 mbar and flow to 20 mL/s. Twenty one days after pathogen inoculation, the disease index was investigated according to the method described in standard NY/T 1464.15–2007 issued by Ministry of agriculture of the People’s Republic of China [13], and the control efficacy was also calculated as described in standard NY/T 1464.15–2007.

### Genome sequencing and analysis

Bacterial genomic DNA was extracted using bacterial genomic DNA kit, and whole genome sequencing was performed using the PacBio RSII single molecule real time sequencing technique with a 20-kb SMRTbellTM library at the Biomarker (Beijing, China). Then gene prediction was further performed by Prodigal 2.50 (E-value<10^−5^) [14], and gene annotation was performed by BlastP similarity searches (E-value<10^−5^) against Clusters of Orthologous Groups (COGs) database. And to exactly determine the phylogenetic taxonomy of strain LM2303, the orthologous genes were generated using OrthoMCL 2.0.3 via comparison with 15 closely related genomes, and a Neighbor-Joining phylogenic tree was constructed based on the matrices of single-copy core genes using Tamura-Nei model by MEGA 7.0.

### Genome mining of secondary metabolite gene clusters with biocontrol functions

The antibiotics and secondary metabolite analysis shell (antiSMASH) serves as a comprehensive resource for the automatic genomic identification and analysis of biosynthetic gene clusters of any type, facilitating rapid genome mining of both bacterial and fungal strains [15, 16]. Thereby, secondary metabolite BGCs in LM2303 genome were mining by using antiSMASH 4.1.0, and further aligned using NCBI BlastP against different databases.

### Production and detection of secondary metabolites from strain LM2303

To produce secondary metabolites, strain LM2303 was firstly cultured as described above, and the cell-free supernatant was collected by centrifugation (4 °C, 12000 g, 15 min) followed by adjusting to pH 2.0 with 6 mol/L HCl. After precipitation overnight at 4 °C, the precipitate was collected by centrifugation (4 °C, 12000 g, 15 min) and extracted with methanol at least three times. Then the filtrated methanol extracts were brought together and evaporated to dryness at 40°C under vacuum using rotary evaporator. The products were dissolved in methanol for further analysis.

Further analysis of secondary metabolites was employed by an Ultra high liquid chromatography (UHPLC) system (Thermo Scientific^™^ Dionex Ultimate 3000 UHPLC, Germany) coupled with a high resolution mass spectrometer (MS) (Thermo Scientific^™^ Q Exactive^™^ Orbitrap MS, Germany). And the MS was integrated with a high-energy collision-dissociation chamber (HCD) and an electrospray ionization (ESI) interface. A 5 μL aliquot was injected into Syncronis C_18_ column (100×2.1 mm, 1.7 μm) in the UHPLC system for separation. The mobile phases were H_2_O (A) and CH_3_CN (B), both containing 0.1% Formic acid (v/v), elution gradient was as follows: 95%A/ 5%B to 5%A/95%B, 60min, flow rate at 300 μL/min. The MS instrumental parameters were as follows: high resolution (70000), positive full scan mode (ESI+), mass range of 150–2000 m/z, microscans of 1, AGC target of 3×10^6^, maximum IT of 100 ms, sheath gas of 30, auxiliary gas of 5, spray voltage of 3.5KV, capillary temperature of 350°C, the interface voltage of 4.5 kv, the detector voltage of 1.2 kv, the desolvation gas temperature of 300 °C, the heat block temperature of 400 °C, the HCD collision energy of 45 eV, nebulizer gas of Nitrogen, flow rate of nebulizer gas of 3 L/min.

## Results and discussion

### Field trial result

Field trial showed the FHB disease index of blank control (sterile water) and fungicide control (Carbendazim) was (55.6±2.9) and (39.5±3.2) respectively, while that of strain LM2303 was only (15.4±1.8), significantly decreased by 71.9% to that of the blank control and 60.5% to that of the fungicide control. Accordingly, the control efficacy of FHB by strain LM2303 was 72.3%, a 43.3% significant increase compared with the fungicide control (*P*<0.01). Thus, strain LM2303 was proved to be a strong potential BCA to prevent wheat from FHB.

### Genome features and analysis of strain LM2303

A circular chromosome of strain LM2303 was obtained (Genbank accession number CP018152), with the length of 3989393 bp, G+C content of 46.68%, and 3866 predicted protein-coding genes. And 2889 of the 3866 protein-coding genes were assigned to one or more COG functional categories (S1 Table). More importantly, compared to the three well-known biocontrol strains (FZB42, CAU B946, M75), strain LM2303 harbored the largest number of genes/gene clusters associated with secondary metabolite biosynthesis, transport, and catabolism (119 genes, 3.08% of the whole genome), amino acid transport and metabolism (349 genes, 9.03% of the whole genome), carbohydrate transport and metabolism (252 genes, 6.51% of the whole genome), inorganic ion transport and metabolism (212 genes, 5.48% of the whole genome)(S1 Table), which indicate that this strain has a higher potential as a biocontrol agent than other *B*. *velezensis* strains.

### Phylogenetic analysis of strain LM2303

**Fig 1:**
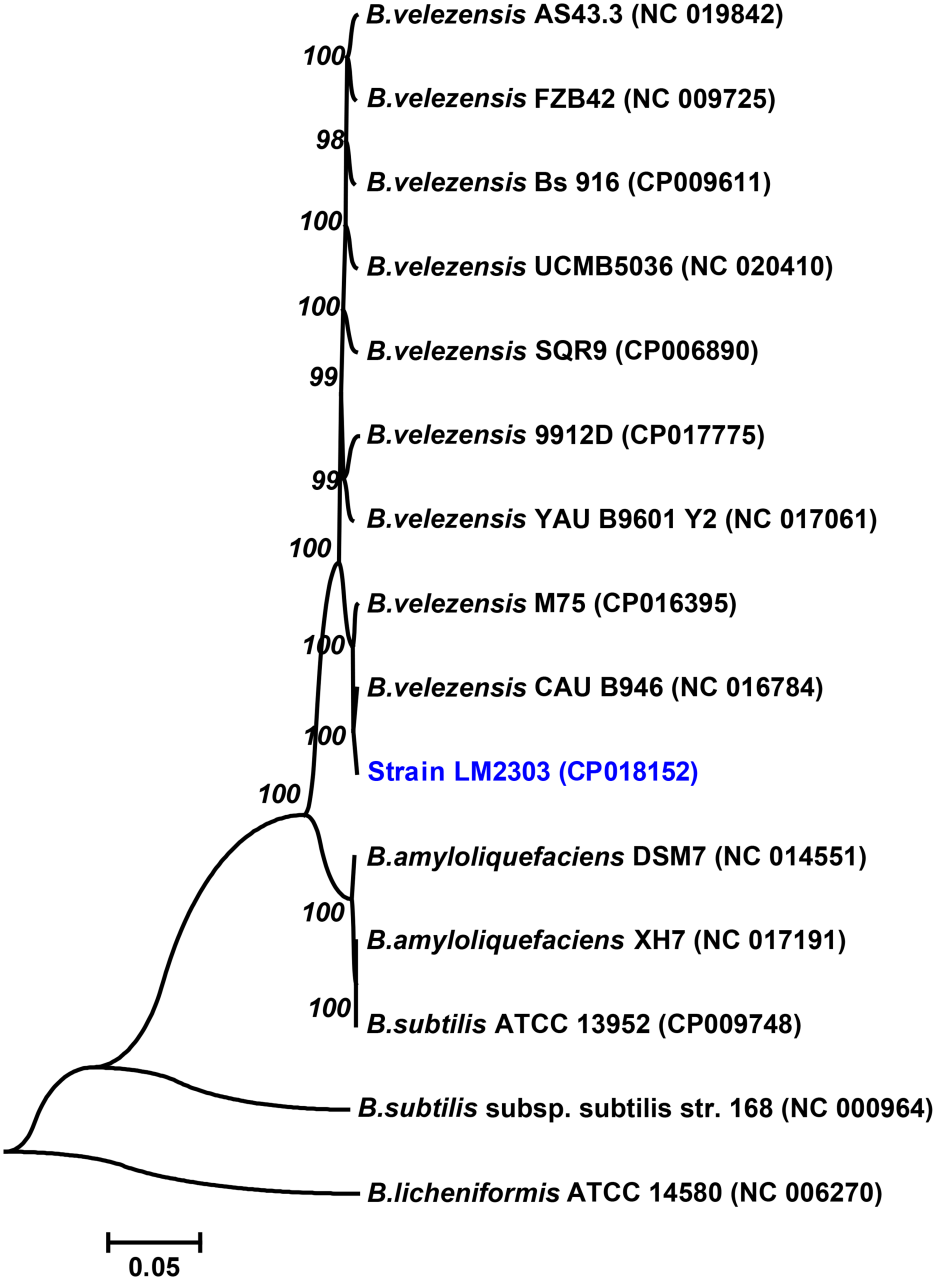
Neighbour-joining phylogenetic tree of strain LM2303. The tree was constructed using the Neighbour-joining method by MEGA 7.0based on 2351 single-copy core genes from 15 related strains with 1500 replications in bootstrap test. Bar, 0.05 nucleotide substitutions per site.

Originally, strain LM2303 was classified as *B*. *subtilis* based on the analysis of morphological, physiological properties and 16S rRNA gene sequences [17]. But, due to the similar morphological, physiological properties and 16S rRNA gene sequences, *B*. *subtilis*, *B*. *amyloliquefaciens*, and *B*. *velezensis* were difficult to differentiate, leading to several re-classifications [18]. In this study, based on the matrices of 2351 single-copy core genes from 15 related strains, strain LM2303 belonged to a different branch with *B*. *subtilis* strain and *B*. *amyloliquefaciens* strain in the phylogenetic tree, but shared the closest evolutionary relative to the *B*. *velezensis* strain (CAU B946 and M75) (Fig 1). Thus strain LM2303 should be a member of the *B*. *velezensis* clade, rather than the *B*. *subtilis* clade or *B*. *amyloliquefaciens* clade. *B*. *velezensis* was first described by Ruiz-Garcia et al. in 2005 and closely related to *B*. *subtilis* and *B*. *amyloliquefaciens* [19]. Until recently, by means of genome sequencing and comparative genomics analysis, several strains including *B*. *amyloliquefaciens* subsp. *plantarum*, *B*. *methylotrophicus* have been reclassified as *B*. *velezensis*, such as the well-known strain FZB42 [18], thereby the taxonomic status of some related strains may need to be reconsidered.

### Secondary metabolite biosynthetic gene clusters in the LM2303 genome

Through genome mining, a total of 29 putative BGCs were found in the LM2303 genome, and 13 of which were identified, including 4 non-ribosomal peptide synthetases (NRPSs) for surfactin, fengycin, bacilysin and bacillibactin, 3 polyketide synthases (PKSs) for difficidin, macrolactin and butirosin, 2 PKS-NRPS hybrid synthetases (PKS-NRPS hybrid) for iturin and bacillaene, 1 Microcin synthase for plantazolicin, 1 Thiopeptide synthase for kijanimicin, 2 Cf_putative synthases for molybdenum cofactor and teichuronic acid. Notably, the 13 annotated BGCs span more than 735 kb and represent nearly 18% of the whole genome, and the metabolites were reported to play important roles in pathogen suppression, nutrient uptake, plant colonization or induced systemic resistance (ISR)(Table 1)[3, 7, 20, 21], accentuating the strong potential of strain LM2303 in the biocontrol application. Besides, the synthetase genes in the LM2303 genome for bacillaene, bacillibactin, bacilysin, difficidin, fengycin, macrolactin showed 100% similarity to those in other three *B*. *velezensis* strains, while the synthetase genes for surfactin showed an obvious difference in the four strains (Table 1). Interestingly, the LM2303 genome contained a fragment of the kijanimicin operon which was absent in FZB42 and CAU B946, and the plantazolicin operon which was absent in M75, endowing strain LM2303 with additional activity against some bacterial pathogens (Table 1). Furthermore, strain LM2303 also harbors another 16 unannotated BGCs (data not shown), which was failed to find similar clusters in the MIBiG database due to less similarity to the existing compounds. The metabolites encoded by the unannotated BGCs may be novel in structure or function, which are worthy to be researched.

**Table 1:** Comparison of secondary metabolite biosynthetic gene clusters in the *B*. *velezensis* strain.

Cluster	Synthetase Type	Metabolite	Function	MIBiG accession^a^	Strains (genes similarity)			
					LM2303	FZB42	CAU B946	M75
*bae*	PKS-NRPS	Bacillaene	Antibacterial	BGC0001089	100%	100%	100%	100%
*dhb*	NRPS	Bacillibactin	Nutrient uptake	BGC0000309	100%	100%	100%	100%
*bac*	NRPS	Bacilysin	Antibacterial	BGC0001184	100%	100%	100%	100%
*btr*	PKS	Butirosin	Antibacterial	BGC0000693	7%	7%	7%	7%
*dif*	PKS	Difficidin	Antibacterial	BGC0000176	100%	100%	100%	100%
*fen*	NRPS	Fengycin	Antifungal	BGC0001095	100%	100%	100%	100%
*itu*	PKS-NRPS	Iturin	Antifungal	BGC0001098	53%	53%	53%	53%
*kijs*	Thiopeptide	Kijanimicin	Antibacterial	BGC0000082	4%	/	/	4%
*mln*	PKS	Macrolactin	Antibacterial	BGC0000181	100%	100%	100%	100%
*moe*	Cf_putative	Molybdenum cofactor	Nutrient uptake	BGC0000916	17%	11%	11%	11%
*pzn*	Microcin	Plantazolicin	Antibacterial	BGC0000569	91%	100%	41%	/
*srf*	NRPS	Surfactin	Antifungal, Antibacterial, Colonization, ISR	BGC0000433	82%	95%	82%	82%
*tua*	Cf_putative	Teichuronic acid	Nutrient uptake	BGC0000868	87%	100%	100%	100%

^a^, Biosynthetic gene cluster ID in the MIBiG database

### Antifungal secondary metabolites from strain LM2303

**Fig 2:**
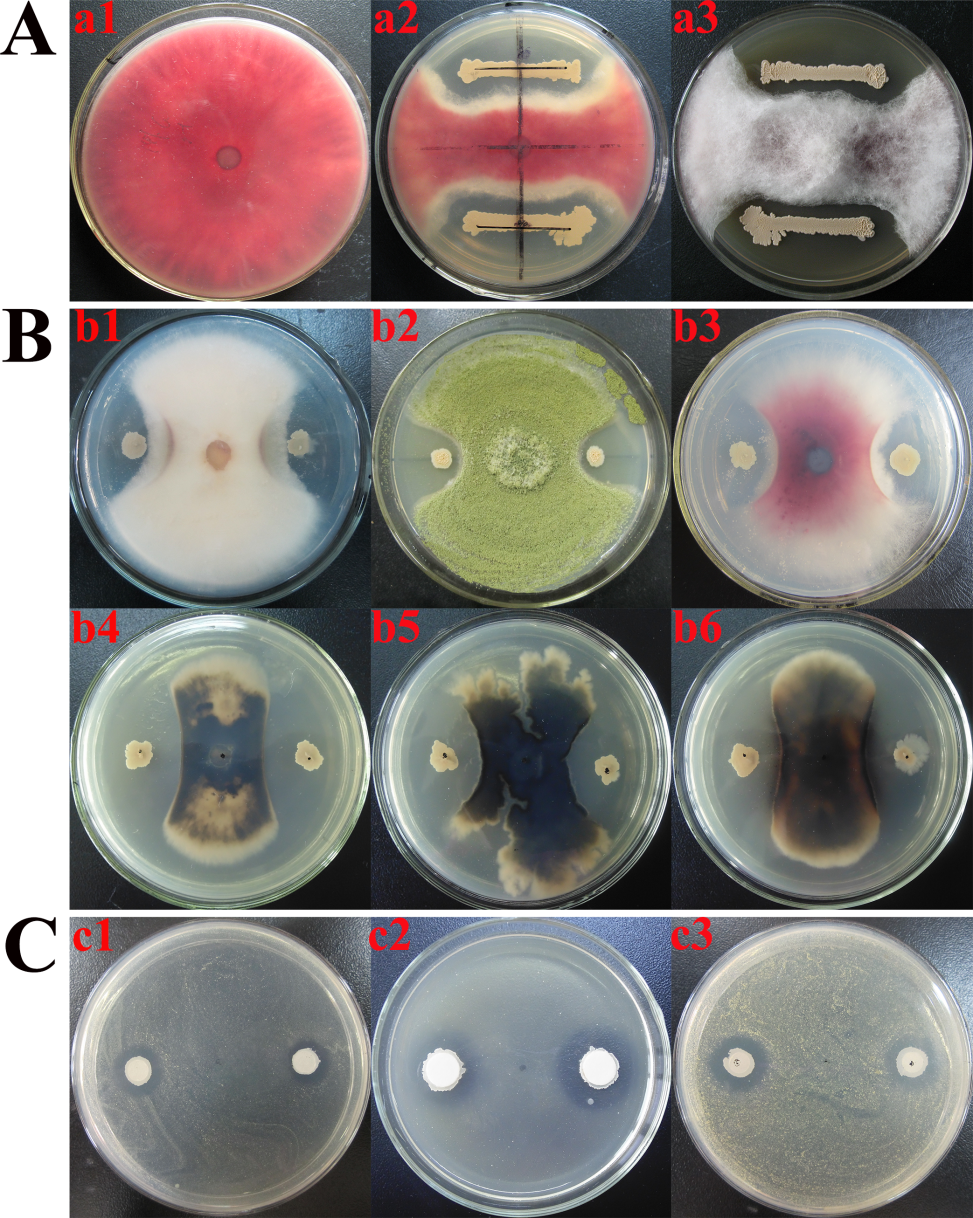
Antimicrobial activity of strain LM2303 against various pathogenic microbes. a1: a 6-mm agar plug of *F*. *graminearum* is inoculated on the center of PDA plate for 6d at 28°C. a2-3: strain LM2303 is simultaneous inoculated with 3cm apart from the plug of *F*. *graminearum*. b1: *F*. *culmorum*, b2: *A*. *flavus*, b3: *F*. *moniliforme*, b4: *Coniothyrium olivaceum*, b5: *Rhizomorpha Roth*. ex Fr, b6: *Alternaria tenuissima*, c1: *X*. *campestris*, c2: *Staphylococcus aureus*, c3: *Sarcine luted*.

**Fig 3:**
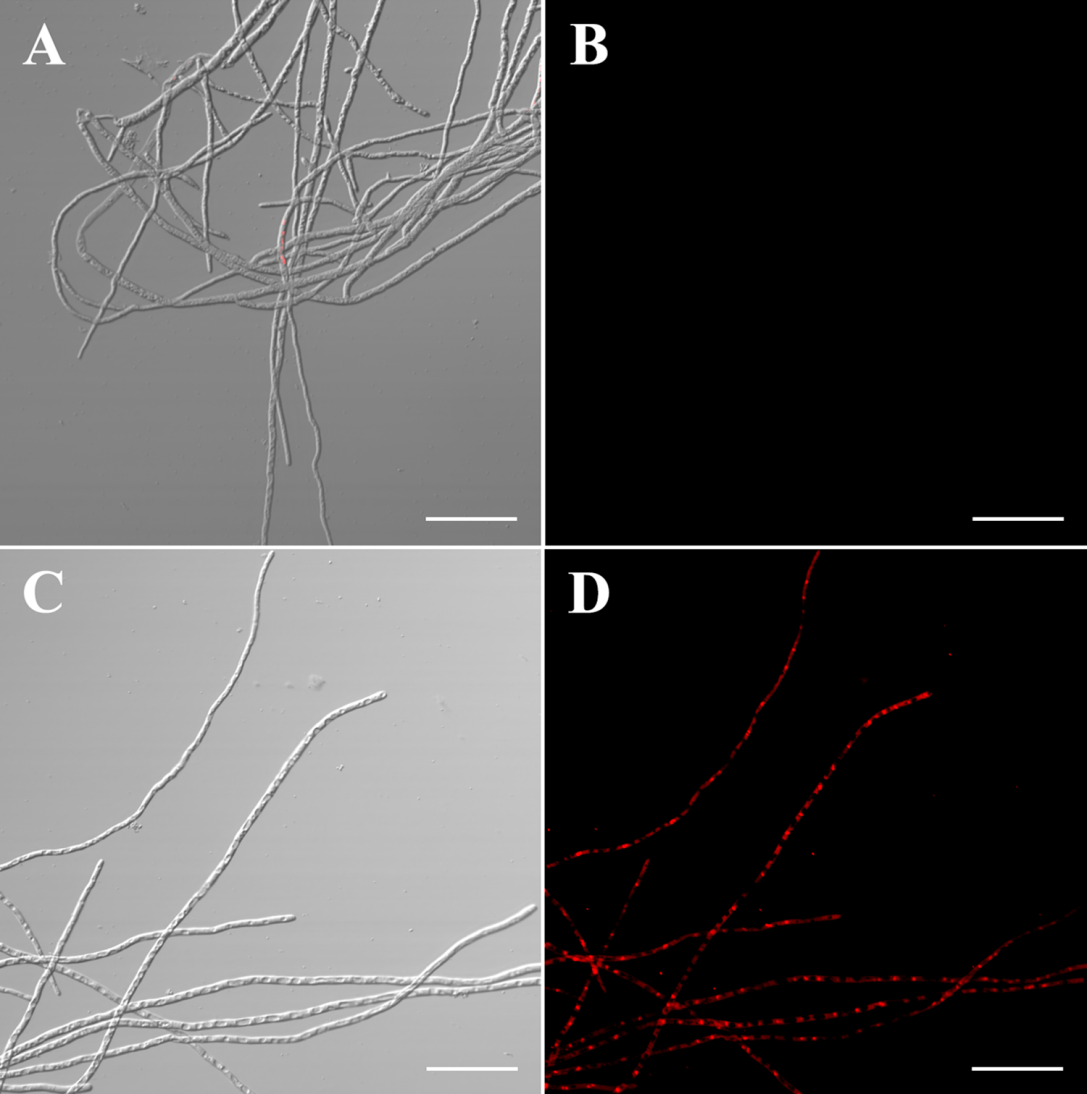
Fuorescence microscopy analyses of *F*. *graminearumon* hyphae treated with LM2303 CLPs. (A) untreated hyphae under white light. (B) untreated hyphae under green fluorescence. (C) treated hyphae under white light. (D) treated hyphae under green fluorescence. Hyphae were treated with 200 mg/mL LM2303 lipopeptides for 4h min at 28°C. All observations were performed by using confocal laser scanning microscopy (Olympus FV1200). Bar = 50μm. Propidium iodide is a popular red-fluorescent nuclear and chromosome counterstain, which is not permeant to normal cell membranes. So PI was used at 1mg/mL as a vital stain based on visualizing membrane permeability.

Strain LM2303 harbors 3 annotated BGCs encoding antifungal metabolites (fengycin, iturin, surfactin) (Table 1), while fengycin, iturin, surfactin are both belong to *Bacillus* cyclic lipopeptides (CLPs). *Bacillus* CLPs were known to have strong antifungal activity on phytopathogenic fungi by penetrating cell membranes, forming ion pore channels, causing membrane osmotic imbalance and even cell death [7]. Strain LM2303 exhibited a directly antagonistic effect against *F*. *graminearum* (Fig 2A), in which the CLPs play an important role. By using PI staining, the red fluorescence was observed in the *F*. *graminearumon* hyphae which was treated with LM2303 CLPs, while the red fluorescence was absent in the normal hyphae, indicating the damage of membrane permeability in the treated hyphae (Fig 3). In addition, strain LM2303 also showed a broad-spectrum antifungal activity towards various phytopathogenic fungi include including *F*. *culmorum*, *Aspergillus flavus*, *F*. *moniliforme*, *Coniothyrium olivaceum*, *Rhizomorpha Roth*. *ex Fr*, *and Alternaria tenuissima* (Fig 2B).

**Fig 4:**
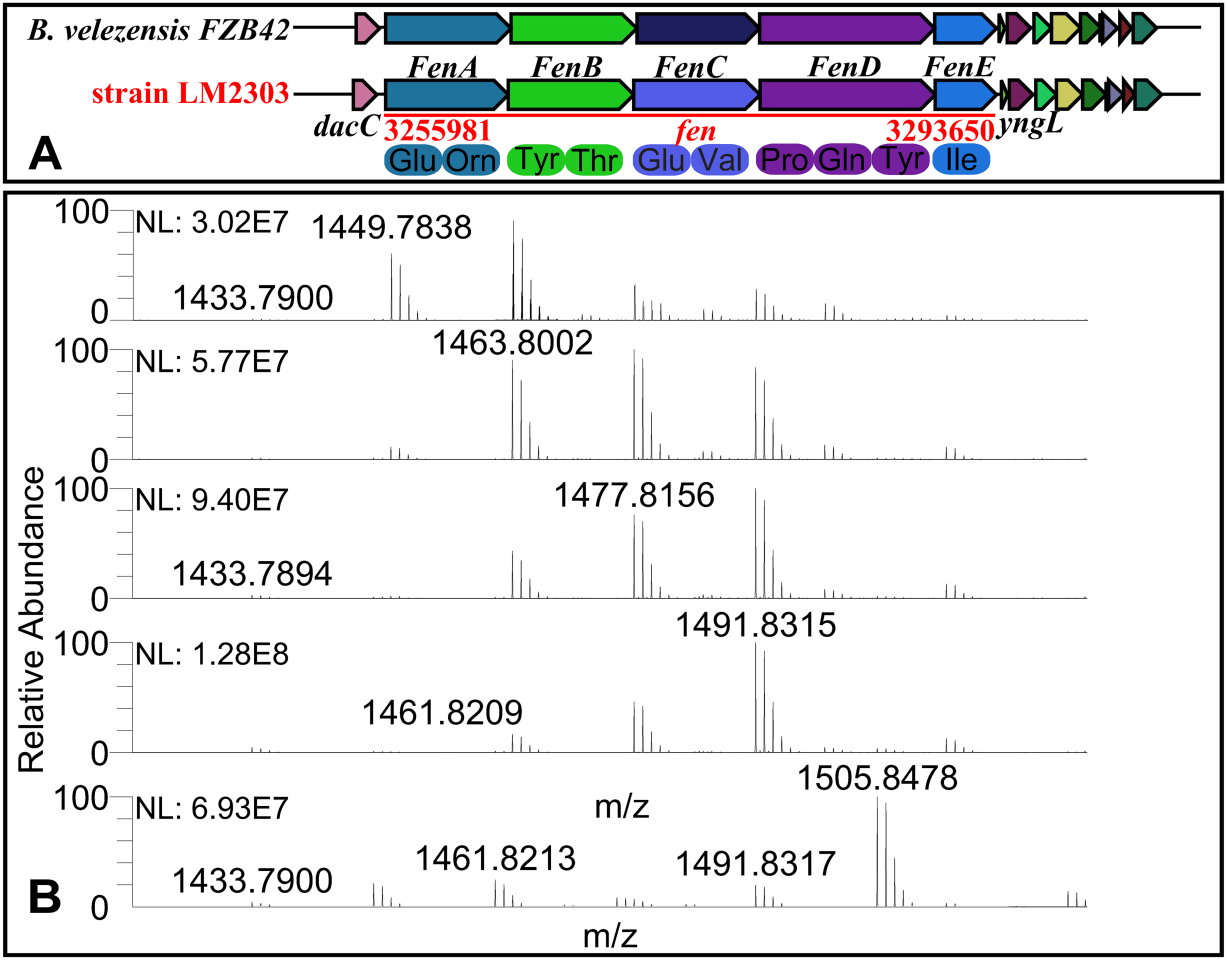
The biosynthetic gene cluster and MS analysis of Fengycin A in strain LM2303. Ions of *m/z* values 1449.7838, 1463.8002, 1477.8156, 1491.8315 and 1505.8478 were assigned to C_13-17_ Fenygcin B [M+H]^+^, repectively.

**Fig 5:**
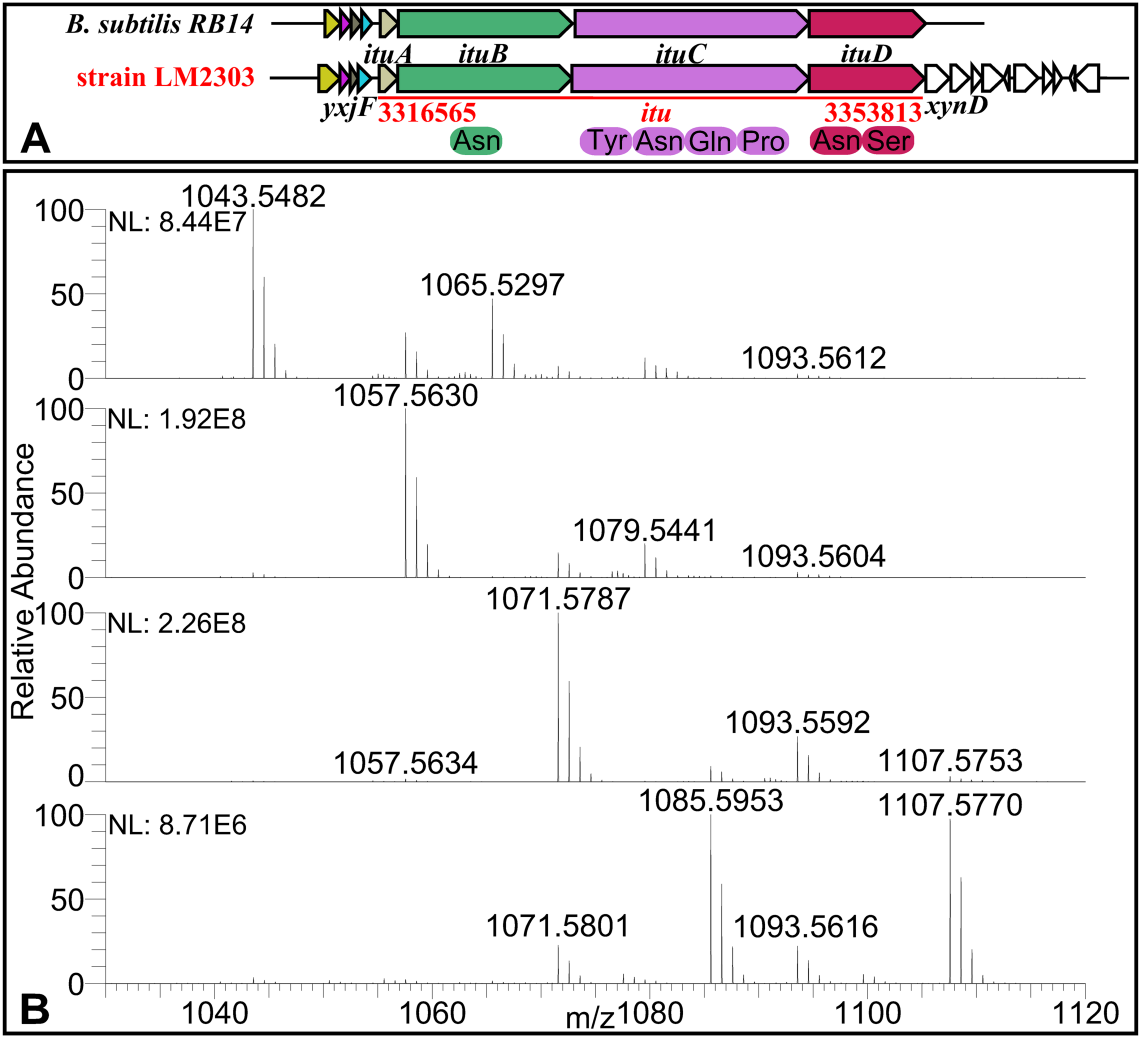
The biosynthetic gene cluster and MS analysis of Iturin A in strain LM2303. Ions of *m/z* values 1043.5482, 1057.5630, 1071.5787 and 1085.5953 were assigned to C_14-17_ Iturin A [M+H]^+^, and ions of *m/z* values 1065.5297, 1079.5441, 1093.5592 and 1107.5570 were assigned to C_14-17_ Iturin A [M+Na]^+^, repectively.

**Fig 6:**
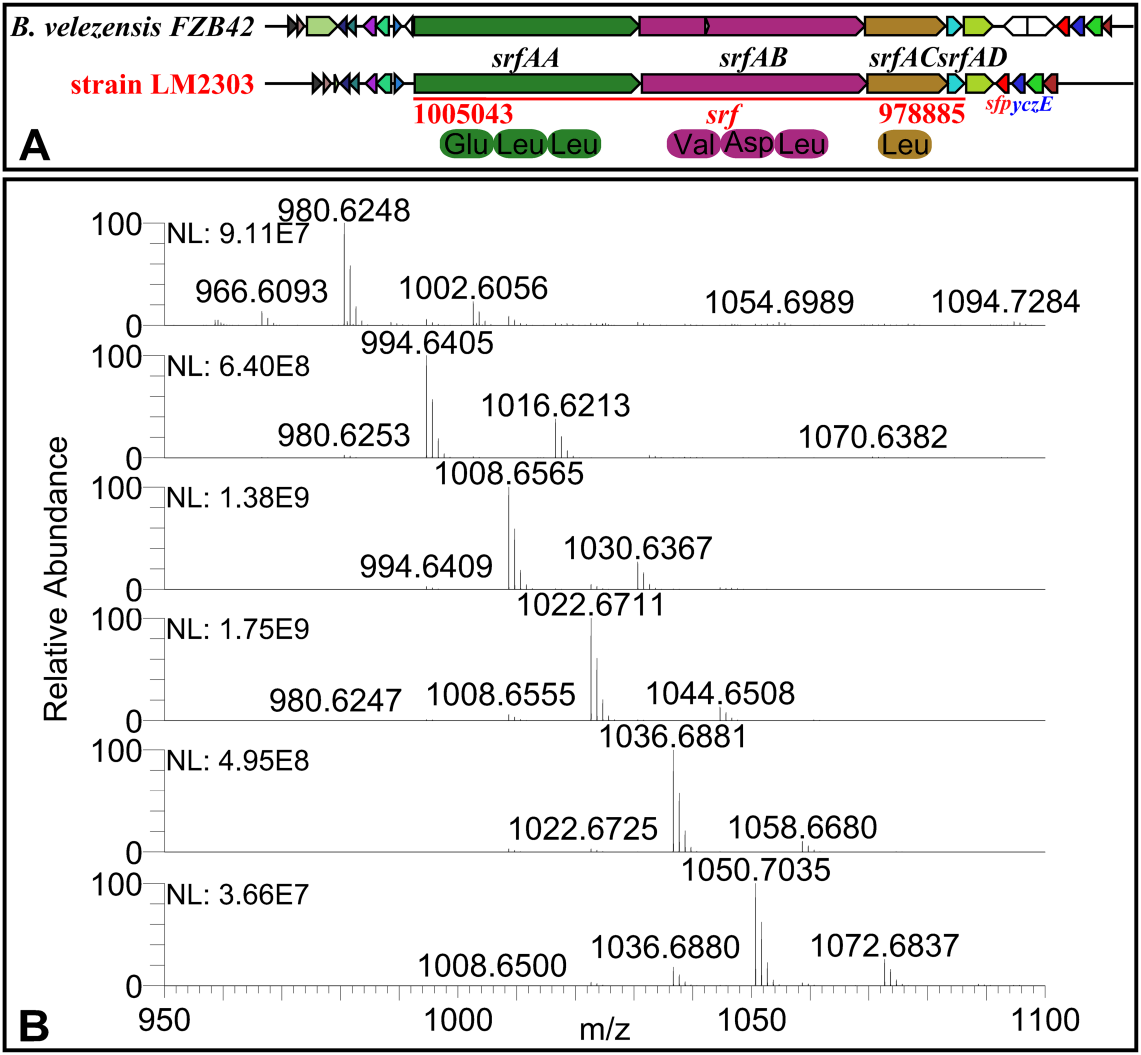
The biosynthetic gene cluster and MS analysis of Surfactin A in strain LM2303. Ions of *m/z* values 994.6405, 1008.6565, 1022.6711, 1036.6881 and 1050.7035 were assigned to C_12-17_ Surfactin A [M+H]^+^, and ions of *m/z* values 1002.6056, 1016.6213, 1030.6367, 1044.6508, 1058.6680 and 1072.6837 were assigned to C_12-17_ Surfactin A [M+Na]^+^, repectively.

Fengycin, recognized strong antifungal activity against filamentous fungi [7], is synthesized by NRPSs encoded by a 37.7-kb gene cluster in LM2303, which shows 100% similarity to the *fen* cluster in FZB42). The *fen* cluster in LM2303 consists of five genes (*fenA*-*E*), both directly involving in non-ribosomal peptide synthesis. The first three genes each encode two amino acid modules, the fourth gene (*fenD*) encode three modules, and the last gene (*fenE*) harbors one amino acid modules (Fig 4). Ions of *m/z* values 1449.7838, 1463.8002, 1477.8156, 1491.8315 and 1505.8478 were detected in the cuture extracts of strain LM2303 and assigned to C_13-17_ fenygcin B [M+H]^+^ by UHPLC-ESI-MS/MS (Fig 4). Unlike fengycin, iturin was encoded by a PKS-NRPS hybrid cluster, which span 37.3-kb in the LM2303 genome. The cluster is an insertion in the genome locating between gene *yxjF* and *xynD*, as in *B*. *subtilis* RB14, contains four genes (i*tuA*-*D*) (Fig 5)[22]. The *ituD* gene encodes malonyl-CoA transacylase, whose disruption results in a specific deficiency in iturin A production, the next three genes *ituA*, *ituB* and *ituC* are encode the NRPSs that harbor one, four and two amino acid modules, respectively (Fig 5). The ions of *m/z* values 1043.5482, 1057.5630, 1071.5787 and 1085.5953 were observed and assigned to C_14-17_ iturin A [M+H]^+^ (Fig 5). Besides, a 26.2-kb *srf* gene cluster responsible for surfactin biosynthesis was also identified in LM2303. The *srf* cluster contains 4 genes (*srfAA*-*AD*), showing 78% of gene similarity to that of FZB42. In addition, the *sfp* gene encoding 4′-phosphopantetheinyl transferase, an essential enzyme for the non-ribosomal synthesis of lipopeptides and the synthesis of polyketides, was also detected together with the regulatory gene *yczE*, while the *comS* gene embedded within *srfAB* in FZB42 was not found in LM2303 (Fig 6). Ions of *m/z* values 994.6405, 1008.6565, 1022.6711, 1036.6881 and 1050.7035 were detected and assigned to C_12-17_ Surfactin A [M+H]^+^ (Fig 6). Unlike fengycin and iturin, which were well-recognized for strong antifungal activity, surfactin is well-known for its powerful surfactant activity and broad-spectrum of antibacterial and antiviral activities, its direct antifungal activity was just reported in recent years. Surfactin was reported to significantly inhibit the hyphae growth of *Magnaporthe grisea* [23], *F*. *verticillioides* [24], *F*. *moniliforme* [25], and *A*. *niger* [26] through the insertion of the fatty acyl chain into the membrane bilayer, causing a strong destabilization of the membrane and changing the physical properties of the membrane [27, 28]. Besides, a [ΔLeu^6^] surfactin derivative was reported to be able to affect the maintenance of DNA integrity in *F*. *moniliforme* by binding with DNA [25].

More importantly, the coproduction of three families of CLPs by strain LM2303 may provide the coordinated biocontrol action and ecological advantages due to their synergistic interactions in biocontrol practice of plant diseases.

### Antibacterial secondary metabolites from strain LM2303

Strain LM2303 harbors 8 annotated BGCs responsible for antibacterial metabolites including surfactin, butirosin, plantazolicin, kijanimicin, bacilysin, difficidin, bacillaene and macrolactin (Table 1). And this strain exhibited a broad-spectum antibacterial activity against Gram-negetive bacteria *X*. *campestris*, Gram-positive bacteria *Staphylococcus aureus*, *Sarcine luted* (Fig 2C).

**Fig 7:**
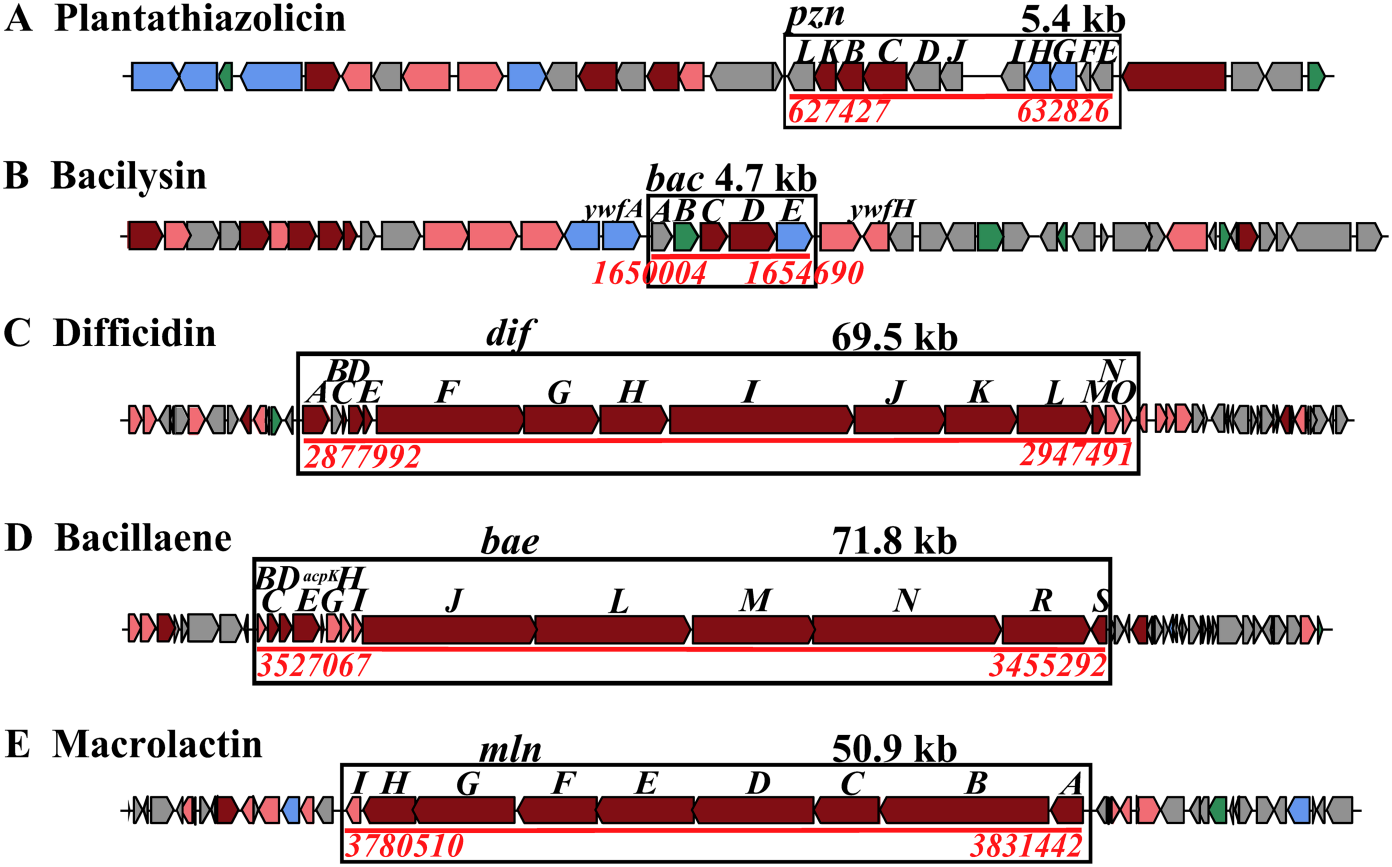
Secondary metabolite gene clusters with antibacterial metabolites in strain LM2303. The above gene clusters were identified by antiSMASH 4.0.0rc1 and further Blastp alignment. The biosynthetic gene cluster with single letter codes above each ORF.

Surfactin was reported to have a broad-spectrum of antibacterial activity, and to significantly inhibit the bacterial diseases in plants, such as Arabidopsis root infection by *Pseudomonas syringae* [29], tomato bacterial wilt caused by *Ralstonia solanacearum* [30]. In addition, another 7 BGCs responsible for other antibacterial metabolites were identified, including butirosin, plantazolicin, kijanimicin, bacilysin, difficidin, bacillaene and macrolactin (Table 1). These BGCs for plantazolicin, bacilysin, difficidin, bacillaene, and macrolactin in LM2303 were found to show high gene similarity to that of FZB42, respectively (Fig 7). Notably, the BGC for kijanimicin presented in LM2303 was absent in FZB42.

**Fig 8:**
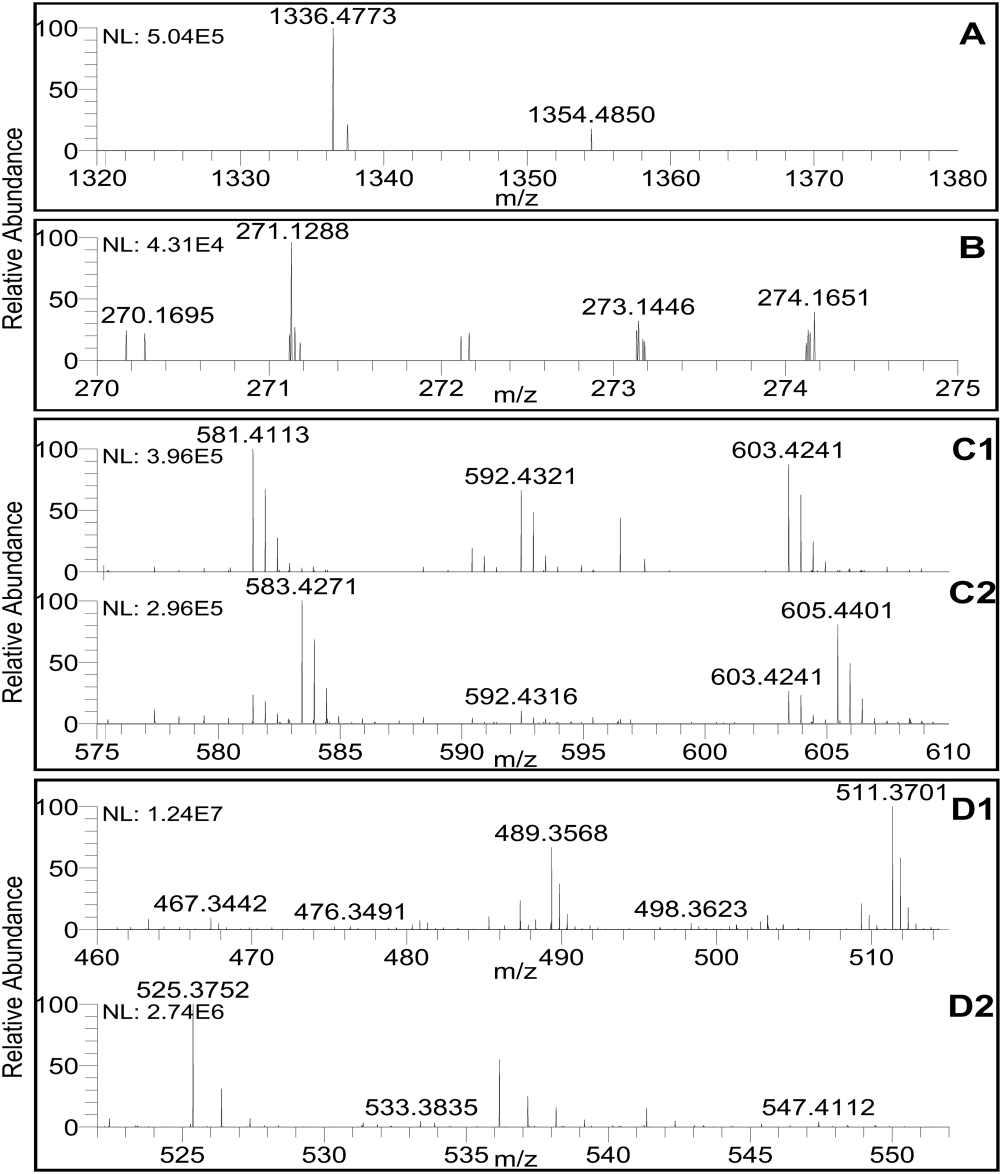
MS analysis of antibacterial secondary metabolites from strain LM2303. (A) *m/z* 1336.4773: plantazolicin [M+H]^+^, *m/z* 1354.4850: hydrolyzed plantazolicin [M+H]^+^; (B) *m/z* 271.1288: Bacilysin [M+H]^+^; (C1) *m/z* 581.4113: Bacillaene A [M+H]^+^, *m/z* 603.4241: Bacillaene A [M+Na]^+^; (C2) *m/z* 583.4271: Bacillaene B [M+H]^+^, *m/z* 605.4401: Bacillaene B [M+Na]^+^; (D1) *m/z* 489.3568: 7-o-malonyl macrolactin A [M+H]^+^, *m/z* 511.3701: 7-o-malonyl macrolactin A [M+Na]^+^; (D2) *m/z* 525.3752: 7-o-succinyl macrolactin A [M+Na]^+^.

Plantazolicin, originally obtained from strain FZB42, was classified as thiazole/oxazole-modified microcin, displaying antibacterial activity toward closely related Gram-positive bacteria such as *B*. *anthracis* [31] and antagonistic effect against nematodes [32]. A 5.4-kb ribosomally encoded cluster containing 11 genes, was identified in LM2303 (Fig 7). As the hydrolytic instability of plantazolicin, ions corresponding to plantazolicin ([M+H]^+^ = *m/z* 1336.4773) and hydrolyzed plantazolicin ([M+H]^+^ = *m/z* 1354.4850) were both detected and verified by comparison with MS data from FZB42 (Fig 8) [33]. Bacilysin, a dipeptide antibiotic with a broad-spectrum antibacterial activity [34], was also synthesized by NRPSs, but not depend on the *sfp* gene. A 4.7-kb gene cluster (*bacA*-*E*), located between gene *ywfH* and *ywfA* as in FZB42, direct the synthesis of bacilysin in LM2303 (Fig 7). And the ion of *m/z* values 271.1288 was observed in the culture extracts of strain LM2303 (Fig 8), which was identical to the calculated m/z of bacilysin [M+H]^+^. Bacilysin has been shown to cause the damages of cell wall in the genus *Xanthomonas* which can infect at least 350 different plants, such as wheat bacterial leaf streak caused by *X*. *translucens* [34–36]. Difficidin was originally isolated from the fermentation broth of a *B*. *subtilis* strain, displaying the broad-spectrum antibacterial activity [37]. It was synthesized by PKSs encoded by a 69.5-kb gene cluster containing 15 genes (*dif A*-*O*) in LM2303 (Fig 7), but both difficidin and its hydroxylated derivatives were not detected in the culture extracts of strain LM2303, the culture condition or assay method may have to be changed. Difficidin has been showed to be efficient in suppressing plant pathogenic bacterium *X*. *oryzae* [34] and *Erwinia amylovora*[38]. Bacillaene, a novel inhibitor of procaryotic protein synthesis, was also originally isolated from the fermentation broth of a *B*. *subtilis* strain, inhibiting both Gram-positive and Gram-negative bacteria [39]. In the LM2303 genome, bacillaene was synthesized by a PKS-NRPS hybrid cluster consisting of 14 genes (*bacb-E*, *acpK*, *bacG-H*) (Fig 7). The presence of bacillaene was also confirmed by UHPLC-ESI-MS, and ions corresponding to bacillaene A ([M+H]^+^ = 581.4113, [M+Na]^+^ = 603.4241) and bacillaene B ([M+H]^+^ = 583.4271, [M+Na]^+^ = 605.4401) were observed (Fig 8)[20]. Besides difficidin and bacillaene, the third polyketide macrolactin, was synthesized by a 50.9-kb *mln* cluster in LM2303. Macrolactin compounds (macrolactin A, 7-O-malonyl macrolactin A and 7-O-succinyl macrolactin) were reported to effectively inhibit the soilborne plant pathogenic bacteria *Ralstonia solanacearum*[40]. Ions corresponding to 7-o-malonyl macrolactin A ([M+H]^+^ = 489.3568, [M+Na]^+^ = 511.3701) and 7-o-succinyl macrolactin A ([M+Na]^+^ = 525.3752) were detected in the culture extracts of strain LM2303 (Fig 8).

In addition, butirosin is a clinically aminoglycoside antibiotic with antibiotic activity against Gram-negative bacteria as well as antiviral activity [41]. Kijanimicin is a spirotetronate antibiotic with antibiotic activity against Gram-positive bacteria as well as antitumor activity [42]. The two metabolites have exhibited the established medicinal activities, but the roles in biocontrol applications have yet to be established. To clarify, although these antibacterial secondary metabolites did not play a direct role in the control of FHB, they would enhance resistance to bacterial diseases in wheat and favor for the normal growth of Wheat.

### Plant colonization

Efficient colonization on plant tissues, is a prerequisite for the biocontrol strains to survive, suppress plant disease or promote plant growth, while the process relies on the surface motility and efficient biofilm formation of bacteria cells [43–45].

**Fig 9:**
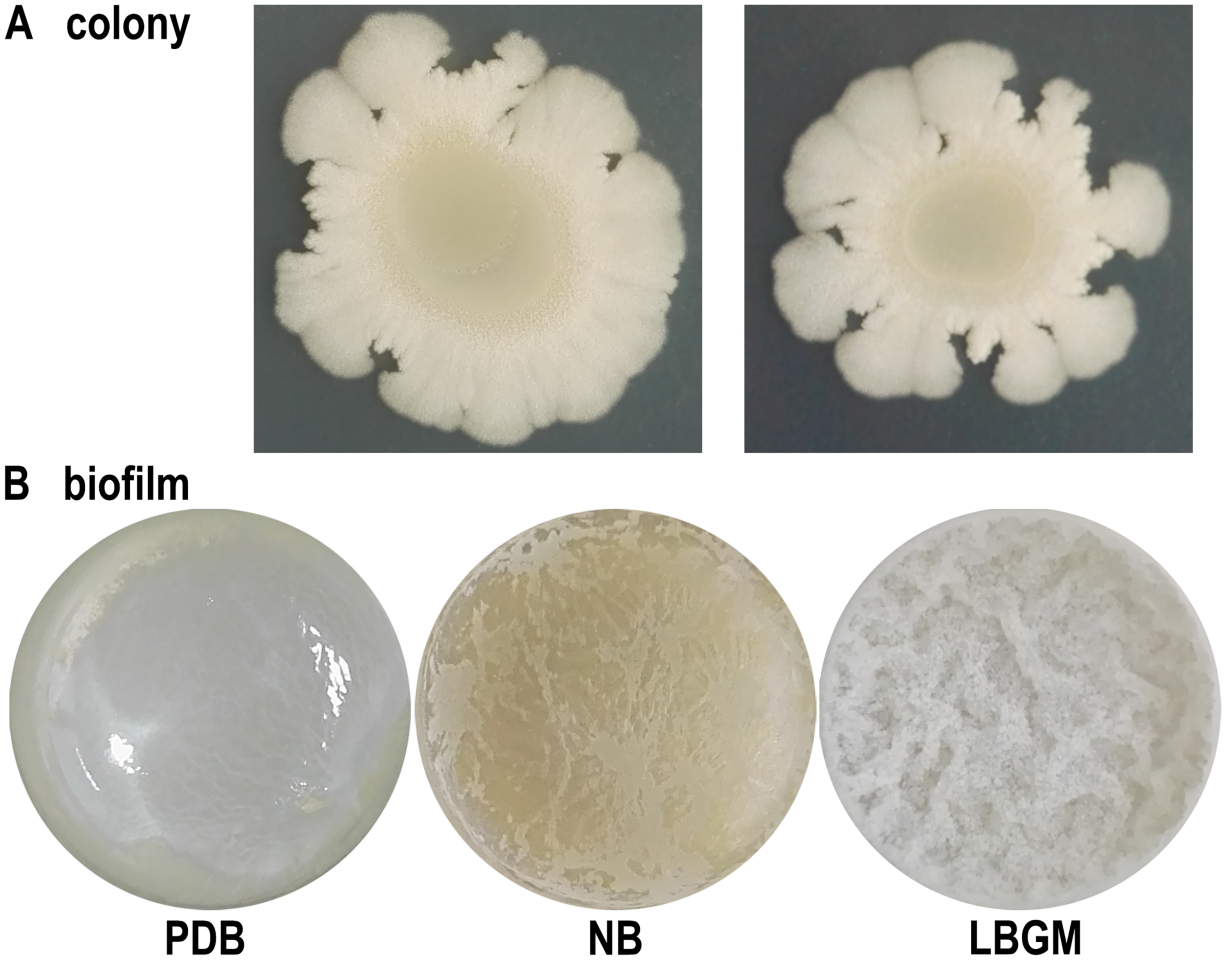
Colonial morphology and biofilm of strain LM2303. (A) Colonial morphology of strain LM2303 in NA, (B) biofilm of strain LM2303 under different culture conditions.

The genes/gene clusters for flagellar assembly (*flg* cluster, *fli* cluster), bacterial chemotaxis (*che* cluster) as well as regulatory gene *swrAA*, *swrB*, *swrC* were both found in the LM2303 genome, and strain LM2303 did exhibit good swarming motility (Fig 9). So it is conceivable that strain LM2303 can actively reach the plant surface through passive movement or flagellum active swimming. The next important step of efficient colonization for biocontrol strains is to enable the formation of bacterial biofilm, which was formed by a variety of extracellular matrix including proteins, polysaccharides, charged polymers and amphiphilic molecules [43]. The *yqxM*-*sipW*-*tasA* operon encoding the *TasA* protein of biofilm, the regulator gene *sinR* and *sinI*, and the *eps* cluster encoding the exopolysaccharide of biofilm, were both found in the LM2303 genome (Table 2). Moreover, a 2.8-kb *pgs* cluster responsible for the synthesis of poly-γ-glutamic acid (PGA), which is a main charged polymer for biofilm, was also found. Meanwhile, the production of PGA by strain LM2303 was confirmed by using the HPLC method as compared with L-glutamic acid standard (date not shown). And the stable biofilm formed by strain LM2303 was observed under different conditions (nutrient broth: NB, potato dextrose broth: PDB, LBGM broth: LBGM (Fig 9). Besides, a positive correlation between biofilm formation and surfactin production was declared, a deficiency in surfactin production led to a defect of biofilm formation and a partial reduction of disease suppression [44]. Moreover, surfactin was also reported to be able to inhibit biofilm formation of pathogenic bacteria including, thus equipping biocontrol strains with powerful antagonistic advantage during colonization and providing plants with a protective barrier [46].

**Table 2:** Genes and gene clusters involved in bacterium-plant interactions in the LM2303 genome.

Bioactivity	Gene/Gene cluster	Product
Biofilm formation	*eps* cluster	Exopolysaccharide component
Biofilm formation	*yqxM*	Uncharacterized protein
	*sipW*	Signal peptidase
	*tasA*	Spore coat-associated protein
	*sinR*, *sinI*	Transcriptional regulator
Biofilm formation	pgs*A-C*	PGA
Nitrogen assimilation	*moaA-E*	Molybdenum cofactor biosynthesis protein A-E
Nitrogen assimilation	*nasD*	Nitrite reductase large subunit
	*nasE*	Nitrite reductase small subunit
	*nrgB*	Nitrogen regulatory protein
	*nrgA*	Ammonium transporter
	*nark*	Nitrate transporter
	*fnr*	Anaerobic regulatory protein
	*arfM*	Probable transcription regulator
	*narG-J*	Nitrate reductase
	*narP*	Nitrate/nitrite response regulator
Iron assimilation	*dhb* cluster	Bacillibactin
Potassium assimilation	*ktrD*	Potassium uptake protein D
	*ktrA*	Potassium uptake protein A
	*ktrC*	Potassium uptake protein C
Bivalent cations assimilation	*tua* cluster	Teichuronic acid
Manganese assimilation	*mntH*	Mn^2+^ transport protein
	*mntC*	Mn^2+^ transport membrane protein
	*mntP*	Mn^2+^ efflux pump
	*mntR*	Transcriptional regulator
Magnesium assimilation	*mgtE*	Mg^2+^ transporter
	*corA*	Mg^2+^/Co^2+^ transport protein
Plant growth promation/ISR (3-hydroxy-2-butanone)	*alsR*	HTH-type transcriptional regulator
	*alsS*	Acetolactate synthase
	*alsD*	Acetolactate decarboxylase
Plant growth promation/ISR	*bdhA*	(R,R)-Butanediol dehydrogenase
Plant growth promation	*phy*	3-Phytase
Plant growth promation (Indole acetic acid)	*ysnE*	N-Acetyltransferase
	*yhcX*	Carbon-nitrogen hydrolase
Plant growth promation (Trehalose)	*treR*	Tre operon transcriptional repressor
	*treA*	Trehalose-6-phosphate hydrolase
	*treP*	Trehalose permease component

### Bacterium-plant interactions

In the biocontrol system, plant, pathogen and biocontrol strain always interact with one another in a variety of different ways, and certain interaction can be triggered by biocontrol strain itself, such as direct antagonism towards pathogens, plant growth promotion, ISR. Plant growth promotion by biocontrol strain can act by making nutrients available for plants and/or producing plant growth-promoting hormones [47]. In the LM2303 genome, the genes/gene clusters related to plant growth promotion were found and summarized in Table 2.

Molybdenum cofactor, a relic of a nitrogen-fixing gene cluster or a cofactor for nitrogen assimilation, is encoded by the *moa* cluster containing 5 genes (*moaA*-*E*) in LM2303 [43, 48]. Moreover, the genes for nitrate transporter (*narK*), anaerobic regulatory protein (*fnr* 1672), probable transcription regulator (*arf*M), nitrate reductase (*narG*-*J*), ammonium transporter (*nrgA*), nitrogen regulatory PII-like protein (*nrgB*), nitrate/nitrite response regulator (*narP*), were also found along with nitrite reductase large subunit (*nasD*), nitrite reductase small subunit (*nasE*), which all favor for nitrogen assimilation. The siderophore bacillibactin, is the third kinds of secondary metabolite synthesized by NRPSs in LM2303, except the CLPs (fengycin, surfactin) and the dipeptide (bacilysin). A 11.7-kb *dhb* cluster containing 5 genes was found in LM2303(Table 2). The presence of bacillibactin is also detected in cuture extracts by UHPLC-ESI-MS, and ions of *m/z* values 883.2607 and 905.2426 were observed and assigned to bacillibactin [M+H]^+^ and bacillibactin [M+Na]^+^ by comparison with previously reported MS data [49]. Siderophores are high affinity Fe^3+^ chelators, thus the presence of siderophore-producing strains can contribute to plant health by complexing iron and making iron less available to pathogenic fungi [45]. Besides, siderophores are also proved to bind stably with many heavy metals such as Cd, Cu, Pb and Zn recently, thus being helpful for plants to relieve soil heavy metals stress in soils [50]. Notably, bacillibactin has been reported to significantly inhibit the growth and invasion of *Phytophthora capsici* and *F*. *oxysporum* and effectively reduce the disease severity [51, 52]. Teichuronic acid, is concerned with the assimilation of bivalent cations and with the maintenance of the negative surface charge carried by the bacterium cells [53]. Its cluster in LM2303 contains 8 genes (*tuaA*-*H*) as in *B*. *subtilis* 168. 3-hydroxy-2-butanone and 2, 3-butendiol, the known volatile plant growth-promoting compounds, can also be produced by strain LM2303. The *als* cluster consisting of 3 genes (*alsR*, *alsS*, *alsD*), encoding enzymes of the biosynthetic pathway from pyruvate to 3-hydroxy-2-butanone, and the gene *bdhA*, encoding enzyme for catalyzing 3-hydroxy-2-butanone to 2,3-butanediol, are both found in LM2303. In addition, strain LM2303 also harbors many genes/gene clusters involved in the biosynthesis of other plant growth-promoting hormones, such as indole acetic acid, trehalose and phytase, which are also summarized in Table 2. And the plant growth-promoting effects of strain LM2303 was confirmed on the early growth of wheat under greenhouse conditions, displaying a significantly increase in seed germination rate (*P*<0.01), shoot length (*P*<0.05) and chlorophyll content of wheat seedling (*P*<0.01) over the un-inoculated control.

In addition, some biocontrol strains can protect plants through ISR, which is in the charge of some specific metabolites secreted by biocontrol strains. Interestingly, 3-hydroxy-2-butanone and 2,3-butendiol, the two well-known plant growth-promoting compounds, were reported to act as the volatile elicitors for ISR identified in *Bacillus spp*., leading to a significantly lower level of disease incidence in *Arabidopsis* [54]. Surfactin was also proved to be the non-volatile and broad-spectrum elicitor for ISR in many *Bacillus* strains [7, 55, 56], providing the ISR-mediated protection against pathogens in plants, e.g. wheat [57], tomato [55]. Further, surfactin was proved to elicit ISR via activation of jasmonate- and salicylic acid-dependent signaling pathways [58]. Besides, C_14_ and C_15_ surfactin were showed to trigger a much stronger defense-inducing activity than those of shorter chain length surfactins [55], and the more surfactins that the strain produces, the stronger the defense-inducing activity is [56].

## Conclusions

*B*. *velezensis* LM2303 showed to significantly reduce the incidence and severity of FHB in wheat under field conditions, with higher biocontrol efficacy than that of chemical fungicide. Ten gene clusters involved in the biosynthesis of antimicrobial metabolites, one gene clusters for the siderophore, and substantial amounts of genes/gene clusters devoted to plant colonization, plant growth promotion, nutrient assimilation, and ISR were identified and confirmed, and their roles in disease control were further clarified.

**Fig 10:**
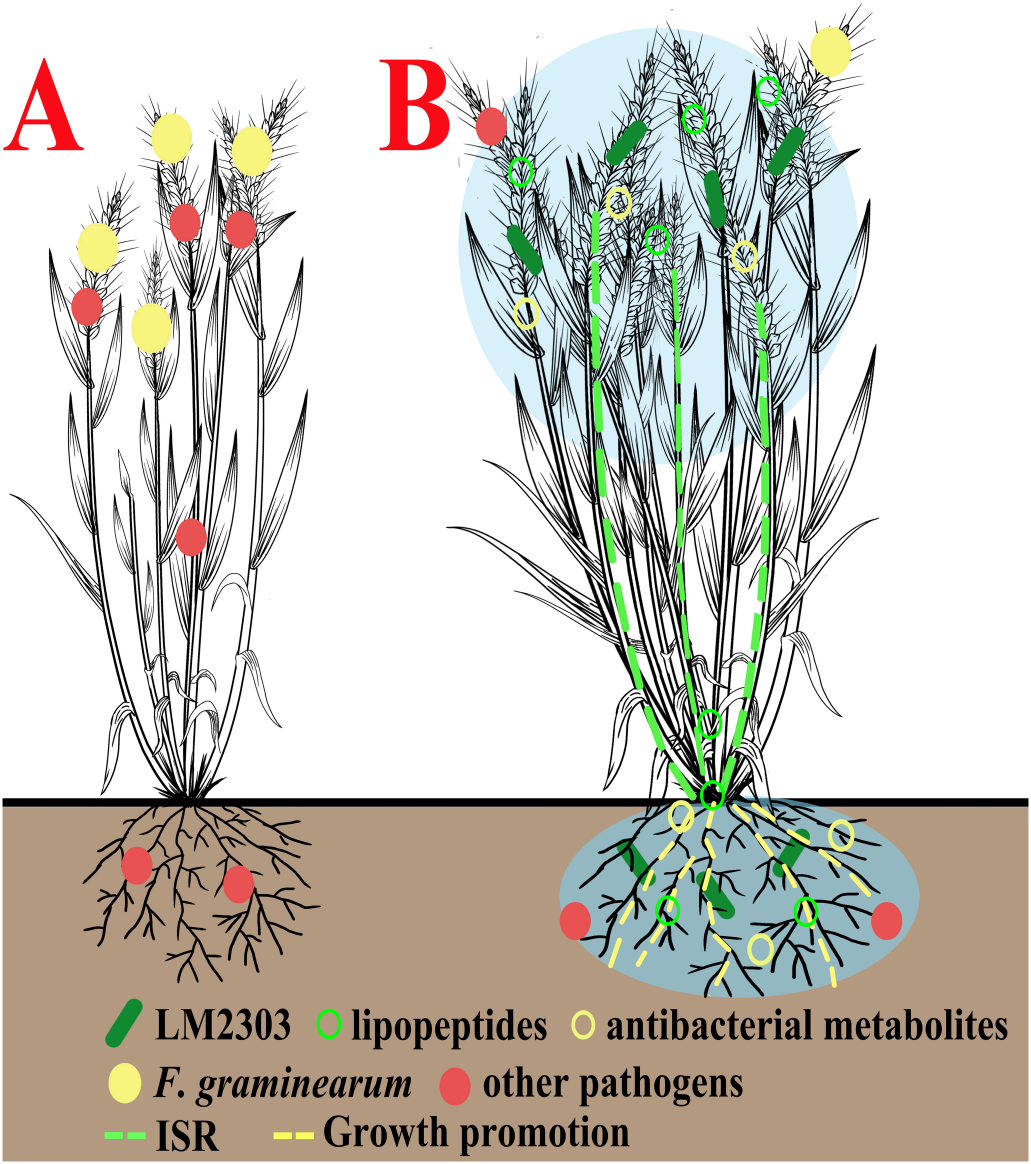
Hypothetical model on the mode of actions of *B*. *velezensis* LM2303 in control of FHB in wheat. (A) an untreated wheat, (B) wheat treated with strain LM2303. The illustration shows our present knowledge about the complex interactions in the tripartite system consisting of biocontrol bacterium (*B*. *velezensis* LM2303, light green rod), pathogen *(F*. *graminearum*, red filled circle) and plant (wheat). Strain LM2303 colonizes the surfaces of wheat tissues relying on its good motility and efficient biofilm formation, and subsequently produces a variety of secondary metabolites so as to form a protective zone (light blue). Cyclic lipopeptides (green circle) act the directly antagonism against *F*. *graminearum* and other pathogenic fungi by inhibit the conidia germination and mycelia growth, and the antibacterial metabolites (yellow *circle)* act the antibacterial activity against wheat bacterial diseases such as black embryo and bacterial leaf streak or alter the bacterial community, which may enhance the survival of strain LM2303. Meanwhile, surfactin and volatiles activate the defense response in wheat, providing an inner ISR-mediated protection against pathogens (green lines). And the promotion of wheat growth (yellow lines) also work effectively by means of nutrient competition and the action of growth-promoting hormones.

Biocontrol mechanisms of plant diseases are varied and complex, and the biocontrol efficacy of microbial strain is the result of complex actions which interact with each other [7, 59]. To our present knowledge, *B*. *velezensis* LM2303 can control FHB in wheat through at least four different mechanisms, as detailed in Fig 10, (i) direct antagonistic action against *F*. *graminearum* and other pathogens mediated by *Bacillus* lipopeptides (green filled circles) and the antibacterial metabolites (yellow filled circles), and (ii) stimulation of ISR in wheat by surfactin and volatiles, and (iii) plant growth promotion by producing growth-promoting hormones and making nutrients available for wheat, e.g., competition for iron through the action of bacillibactin, and (iv) space and nutrient competition via efficient colonization and long-term persistence. Thus, these results suggested that strain LM2303 could be a useful BCA for FHB control.

Future work will focus on the mutagenesis, transcriptomics, and metabolomics of strain LM2303 to improve the biocontrol efficacy, and the response of wheat in simultaneous presence of strain LM2303 and *F*. *graminearum*.

## Supporting information

S1 Table Comparison on COG functional categories of four biocontrol strains. (DOC)
