# Supplementary material for: A comprehensive understanding of the biocontrol potential of Bacillus velezensis LM2303 against Fusarium head blight
Source: PLoS One. 2018 Jun 1;13(6):e0198560. doi: 10.1371/journal.pone.0198560 (PMC5983450; doi:10.1371/journal.pone.0198560)
Supplement: S1 Table — (DOC) [file pone.0198560.s001.doc]

# S1 Table

**Comparison on COG functional categories of four biocontrol strains**

| **COG code** | **Description** | **LM2303** | **FZB42** | **CAU B946** | **M75** |
| --- | --- | --- | --- | --- | --- |
| C | Energy production and conversion | 177 | 169 | 167 | 181 |
| D | Cell cycle control, cell division, chromosome partitioning | 34 | 37 | 36 | 33 |
| E | Amino acid transport and metabolism | 349 | 275 | 286 | 287 |
| F | Nucleotide transport and metabolism | 83 | 82 | 81 | 81 |
| G | Carbohydrate transport and metabolism | 252 | 236 | 232 | 241 |
| H | Coenzyme transport and metabolism | 126 | 120 | 116 | 100 |
| I | Lipid transport and metabolism | 116 | 122 | 121 | 111 |
| J | Translation, ribosomal structure and biogenesis | 161 | 155 | 156 | 156 |
| K | Transcription | 297 | 240 | 247 | 246 |
| L | Replication, recombination and repair | 137 | 112 | 122 | 137 |
| M | Cell wall/membrane/envelope biogenesis | 177 | 174 | 183 | 188 |
| N | Cell motility | 57 | 61 | 62 | 48 |
| O | Posttranslational modification, protein turnover, chaperones | 100 | 97 | 100 | 98 |
| P | Inorganic ion transport and metabolism | 212 | 154 | 149 | 181 |
| Q | Secondary metabolites biosynthesis, transport and catabolism | 119 | 64 | 65 | 87 |
| R | General function prediction only | 452 | 337 | 345 | 290 |
| S | Function unknown | 294 | 318 | 330 | 783 |
| T | Signal transduction mechanisms | 147 | 130 | 122 | 137 |
